# Acute Increase in *O*-GlcNAc Improves Survival in Mice With LPS-Induced Systemic Inflammatory Response Syndrome

**DOI:** 10.3389/fphys.2019.01614

**Published:** 2020-01-21

**Authors:** Josiane Fernandes Silva, Vania C. Olivon, Fabiola Leslie A. C. Mestriner, Camila Ziliotto Zanotto, Raphael Gomes Ferreira, Nathanne Santos Ferreira, Carlos Alberto Aguiar Silva, João Paulo Mesquita Luiz, Juliano Vilela Alves, Rubens Fazan, Fernando Queiróz Cunha, Jose Carlos Alves-Filho, Rita C. Tostes

**Affiliations:** ^1^Department of Pharmacology, Ribeirão Preto Medical School, University of São Paulo, Ribeirão Preto, Brazil; ^2^Universidade Anhanguera-UNIDERP, Campo Grande, Brazil; ^3^Department of Surgery and Anatomy, Ribeirão Preto Medical School, University of São Paulo, Ribeirão Preto, Brazil; ^4^Department of Physiology, Ribeirão Preto Medical School, University of São Paulo, Ribeirão Preto, Brazil

**Keywords:** *O*-GlcNAc, sepsis, LPS, inflammation, vascular reactivity, blood pressure

## Abstract

Sepsis is a systemic inflammatory response syndrome (SIRS) resulting from a severe infection that is characterized by immune dysregulation, cardiovascular derangements, and end-organ dysfunction. The modification of proteins by *O*-linked N-acetylglucosamine (*O*-GlcNAcylation) influences many of the key processes that are altered during sepsis, including the production of inflammatory mediators and vascular contractility. Here, we investigated whether *O*-GlcNAc affects the inflammatory response and cardiovascular dysfunction associated with sepsis. Mice received an intraperitoneal injection of lipopolysaccharide (LPS, 20 mg/Kg) to induce endotoxic shock and systemic inflammation, resembling sepsis-induced SIRS. The effects of an acute increase in *O*-GlcNAcylation, by treatment of mice with glucosamine (GlcN, 300 mg/Kg, i.v.) or thiamet-G (ThG, 150 μg/Kg, i.v.), on LPS-associated mortality, production and release of cytokines by macrophages and vascular cells, vascular responsiveness to constrictors and blood pressure were then determined. Mice under LPS-induced SIRS exhibited a systemic and local inflammatory response with increased levels of interleukin-1β (IL-1β), interleukin-6 (IL-6) and tumor necrosis factor (TNF-α), as well as severe hypotension and vascular hyporesponsiveness, characterized by reduced vasoconstriction to phenylephrine. In addition, LPS increased neutrophil infiltration in lungs and produced significant lethality. Treatment with GlcN and ThG reduced systemic inflammation and attenuated hypotension and the vascular refractoriness to phenylephrine, improving survival. GlcN and ThG also decreased LPS-induced production of inflammatory cytokines by bone marrow-derived macrophages and nuclear transcription factor-kappa B (NF-κB) activation in RAW 264.7 NF-κB promoter macrophages. Treatment of mice with ThG increased *O*-glycosylation of NF-κB p65 subunit in mesenteric arteries, which was associated with reduced Ser^536^ phosphorylation of NF-κB p65. Finally, GlcN also increased survival rates in mice submitted to cecal ligation and puncture (CLP), a sepsis model. In conclusion, increased *O*-GlcNAc reduces systemic inflammation and cardiovascular disfunction in experimental sepsis models, pointing this pathway as a potential target for therapeutic intervention.

## Introduction

Sepsis is a systemic inflammatory response syndrome (SIRS) resulting from a severe infection that is characterized by immune dysregulation, cardiovascular derangements, and end-organ dysfunction. Despite the significant progress in the understanding of the pathophysiologic processes involved in these diseases, their incidence is remarkable. Estimates point to 31.5 million sepsis cases, and approximately 5.3 million deaths per year in high-income countries (Fleischmann et al., 2016). In a Brazilian observational study, the mortality rate of septic patients reached 55% (Machado et al., 2017). These numbers justify the efforts to clarify the systemic alterations during sepsis progress and to improve its recognition and treatment.

During an infection, the host immune system recognizes invading pathogens by identifying pathogen-associated molecular patterns (PAMPs) through a variety of cell-surface and intracellular pattern recognition receptors (PRRs) on the innate immune cells, such as Toll-like receptors (TLRs), nucleotide-binding oligomerization domain-like receptors (NLRs; which activation leads to inflammasome formation), retinoic acid-inducible gene-like receptors (RLRs) and C-type lectin receptors (CLRs) (Takeuchi and Akira, 2010; van der Poll et al., 2017; Salomão et al., 2019). In most cases, the immune system is able to remove the invading pathogen. However, the predominance of the pathogens and exacerbation of host immune response can lead to an excessive inflammatory response, resulting in cardiovascular collapse and organ dysfunction.

Both in the early and late phases of sepsis, the activation of immune cells initiates a complex signal transduction pathway that culminates in the activation of nuclear transcription factor-kappa B (NF-κB) and mitogen-activated protein kinases (MAPK), with consequent release of large amounts of cytokines, chemokines and activation of the complement system (Munoz et al., 1991; Silasi-Mansat et al., 2010; Takeuchi and Akira, 2010; Wiersinga et al., 2014; Salomão et al., 2019). Indeed, macrophages activated by lipopolysaccharide (LPS), the major component of the outer membrane of Gram-negative bacteria, primarily release inflammatory mediators such as interleukin-1β (IL-1β), interleukin-6 (IL-6) and tumor necrosis factor-alpha (TNF-α), as well as secondary mediators involved in pathogens elimination, such as nitric oxide (NO) and reactive oxygen species (ROS) (Ayala et al., 1996; Lu et al., 2015; Seo et al., 2016). The excessive production of NO and ROS is directly involved in vascular damage, leading to excessive vasodilation, edema formation, leukocytes and platelets adhesion, glycocalyx shedding, and endothelial dysfunction (Huet et al., 2011; Tyml, 2011), critical processes in septic condition.

Cellular metabolism has arisen as a key mechanism that modulate inflammatory responses and may play a crucial role in sepsis. In macrophages, the upregulation of glycolysis, for example, increases the transcription of IL-1β, which is related to succinate accumulation and, subsequently, activation of hypoxia-inducible factor 1α (HIF1α) (Lachmandas et al., 2016). Moreover, LPS stimulation of macrophages increases mitochondrial oxidation and ROS generation and promotes a pro-inflammatory state (Cheng et al., 2016). High-glucose also leads to NF-κB activation in vascular smooth muscle cells (Tannahill et al., 2013) and peripheral blood mononuclear cells as observed in diabetic patients (Mills et al., 2016). In this context, the hexosamine metabolism pathway, through the *O*-glycosylation with N-acetylglucosamine (*O*-GlcNAc) of innumerous proteins, can also contribute to the regulation of the inflammatory response. *O*-GlcNAc, a highly dynamic post-translational modification of nuclear and cytoplasmic proteins (Yang and Qian, 2017; Zachara, 2018), is directly controlled by the activity of two enzymes: *O*-GlcNAc transferase (OGT, uridine diphosphate-N-acetyl glucosamine; β-N-acetylglucosaminyl transferase and UDP-NAc transferase) and β-N-acetylglucosaminidase (O-GlcNAcase or OGA). Whereas OGT catalyzes the addition of N-acetylglucosamine in the hydroxyl group of serine and threonine residues of target proteins, OGA catalyzes the hydrolytic removal of *O*-GlcNAc from proteins (Yang and Qian, 2017). Hence, OGT and OGA expression and activity are important to regulate *O*-GlcNAc and, consequently, proteins function by changing their sub-cellular localization, activity, and interaction with other proteins (Andrali et al., 2007; Guinez et al., 2011; Baudoin and Issad, 2015; Zachara, 2018).

In the cardiovascular system, *O*-GlcNAc modulates cardiac and vascular contractility and remodeling as well as inflammation-related processes (da Costa et al., 2018; Kronlage et al., 2019). Glucosamine, which increases *O*-GlcNAc-modified proteins, reduces the levels of circulating pro-inflammatory cytokines, including TNF-α and IL-6 (Azuma et al., 2015) and attenuates LPS-induced activation of NF-κB in RAW264.7 macrophages (Shin et al., 2013). Glucosamine also reduces vascular expression of pro-inflammatory mediators, inhibiting acute inflammatory responses and neointimal layer formation following balloon catheter injury of the carotid artery in ovariectomized rats (Xing et al., 2008), indicating that *O*-GlcNAc has anti-inflammatory and vasoprotective actions.

Monocytes and macrophages play central roles in acute and chronic inflammatory processes. Disturbed metabolism also shifts the profile production of inflammatory mediators by macrophages. For instance, high glucose increases IL-1β, TNFα, TLR2, TLR4, and NF-κB signaling (Shanmugam et al., 2003; Dasu et al., 2008; Hua et al., 2012), contributing to diabetes-associated pro-inflammatory state. On the other hand, stimulation of the hexosamine pathway by glucosamine attenuates LPS-induced NO and NF-κB activation in macrophage cells (Hwang et al., 2017) whereas OGT degradation increases endothelial inflammatory responses induced by hypoxia (Liu et al., 2014). Li et al. (2019) observed that mice with OGT deletion exhibit an exacerbated innate immune response and septic inflammation induced by LPS, events dependent on TLR/RIPK3/NF-κB signaling activation. These results demonstrate that reprogramming of cellular metabolic events can be a fine-tuning point to modulate the inflammatory response in septic conditions. However, it is not clear whether activation of the hexosamine pathway has cardiovascular protective effects in sepsis.

Therefore, considering that *O*-GlcNAc modulates the function of the cardiovascular and immune systems, which are key in endotoxemic/sepsis-associated conditions and complications, this study tested the hypothesis that acute increases in *O*-GlcNAc reduce NF-κB activation and expression/release of pro-inflammatory cytokines, improving cardiovascular function in animals with LPS-induced SIRS.

## Materials and Methods

### Animals

Male C57BL6/J mice (8 to 10-week-old, 20–25 g) were used in this study. Mice were kept in the animal facility of the Department of Pharmacology, Ribeirão Preto Medical School, University of São Paulo, under controlled temperature (22–24°C) and humidity, 12-hour (h) light/dark cycles and received standard diet and water *ad libitum*. All experimental procedures were approved by the Ethics Committee on Animal Research of the Ribeirão Preto Medical School, University of São Paulo (protocol n° 196/13) and are in accordance with the Guidelines of the National Council for Animal Experimentation Control (CONCEA).

### Experimental Animal Models and Pharmacological Tools to Increase *O*-GlcNAc-Modified Proteins

Severe endotoxemia by LPS was induced with a single dose (20 mg/Kg, i.p.) of LPS (Escherichia coli 0111:B4, Sigma Chemical Co., St. Louis, MO, United States), 30 min after treatment with GlcN (300 mg/Kg/animal, i.v.), or 12 h after treatment with ThG (150 μg/Kg; i.v.). Proper controls were performed with vehicle administration (for LPS, GlcN or ThG) at the same time points. To evaluate the effects of acute increased *O*-GlcNAc in mice with endotoxemia induced by LPS, animals were randomly divided into the following groups: saline (Control); vehicle + 20 mg/Kg LPS (LPS); glucosamine or thiamet-G plus 20 mg/Kg LPS (LPS + GlcN and LPS + ThG, respectively). Six hours after LPS treatment, mice were anesthetized with isoflurane and fluids and tissues were collected. The time for GlcN and ThG administration were determined in preliminary protocols (Supplementary Material and Supplementary Figure S1).

Some experiments were also performed in mice that received a lower dose of LPS (10 mg/Kg, i.p.), which induces moderate endotoxemia (LPS_l__o__w_). The following groups were tested in specific protocols: saline (Control); vehicle + 10 mg/Kg LPS (LPS_l__o__w_); glucosamine or thiamet-G plus 10 mg/Kg LPS (LPS_l__o__w_ + GlcN and LPS_l__o__w_ + ThG, respectively). Similarly, GlcN and ThG were administered 30 min and 12 h, respectively, before LPS injection.

Finally, mice with cecal ligation and puncture (CLP)-induced sepsis were used to confirm the effects of GlcN in specific protocols. CLP was induced, as previously described (Alves-Filho et al., 2010). After anesthesia and abdominal wall shaving of the mice, the cecum was exposed through midline laparotomy. The cecum ligation was performed below the ileocecal valve without causing intestinal obstruction and then punctured with a 18G needle. Mice were divided in six groups: (1) Sham, mice submitted to midline laparotomy without cecum ligation and puncture and that received 1 mL of saline i.v.; (2) Sham + hydration + antibiotic therapy, mice submitted to sham operation and that received a single i.v. dose of 1 mL of saline. Antibiotic therapy was performed with sodium Ertapenem (30 mg/Kg, s.c., 1 h) after CLP and every 12 h, for 3 days; (3) Sham + hydration + antibiotic therapy + GlcN treatment, mice submitted to sham operation + hydration + antibiotic therapy + GlcN (300 mg/kg. i.v.); (4) CLP, mice submitted to the CLP procedure; (5) CLP + hydration + antibiotic therapy group (CLP + ATB), CLP mice that received a single dose of 1 mL of saline and were treated with sodium Ertapenem; and (6) CLP + ATB + GlcN, CLP mice submitted to hydration + ATB and that received GlcN.

### Survival Rates

Following treatment of mice with GlcN or ThG and LPS-induced SIRS, the survival rates were determined daily, every 12 h, for 7 days. Survival rates were also determined in mice with CLP-induced sepsis for 7 days. The survival rate is expressed as the percentage of live animals, and the Mantel-Cox log-rank test was used to determine differences between the experimental groups (Alves-Filho et al., 2010).

### Neutrophil Migration to the Peritoneal Cavity

Neutrophil migration was determined as previously described (Alves-Filho et al., 2010). Peritoneal cavity lavage (PCL) was performed 6 h after LPS-induced SIRS or CLP-induced sepsis using 3 ml of phosphate buffer saline/ethylene diamine tetraacetic acid (PBS/EDTA) 1 mM and peritoneal fluids were collected. A Coulter AcT Diff analyzer (Beckman Coulter) was used to perform total cells counts. Differential cell counts were determined on Cytospin slides stained with Panótico Rápido LB dye (Laborclin, Brazil). The results are expressed as the mean number of neutrophils ± SEM per cavity.

### Myeloperoxidase Activity Assay

Lipopolysaccharide or CLP-induced leukocyte migration to the lungs was evaluated using a myeloperoxidase kinetic-colorimetric assay. Tissue samples were collected in 50 mM K_2_HPO_4_ buffer (pH 6.0) containing 13.72 mM hexadecyltrimethylammonium bromide (HTAB) and stored at −80°C until assayed. The samples were homogenized using a Tissue-Tearor (BioSpec), and homogenates were centrifuged (13.000 rpm, 2 min, 4°C). Supernatants were assayed spectrophotometrically for myeloperoxidase activity at 450 nm (Victor3 1420 multilabel counter). Myeloperoxidase activity of samples was compared to a standard curve of neutrophils and is presented as the number of neutrophils per mg of tissue (Alves-Filho et al., 2010).

### Determination of Cytokines Levels

TNF-α, IL-1β, and IL-6 concentrations in cell culture supernatant and serum were determined using enzyme-linked immunosorbent assay (ELISA, R&D Systems, United States) according to the manufacturer’s instructions. Briefly, each well of flat-bottomed 96-well microliter plates was coated with 100 μL of an antibody specific to one of the above cytokines and incubated overnight at 37°C. Plates were washed, and non-specific binding was blocked with 1% bovine serum for 120 min at 37°C. Samples and standards were loaded onto plates. Recombinant murine (rm) TNF-α, IL-1β, and IL-6 standard curves were used to calculate cytokines concentrations. Plates were thoroughly washed, and the appropriate biotinylated polyclonal or monoclonal anti-cytokine antibody was added. After one h, the plates were washed, avidin-peroxidase was added to each well for 15 min, and plates were thoroughly washed again. After addition of substrate, the reaction was stopped with H_2_SO_4_ (1 mol/L), and the optical density of the individual samples was measured on an ELISA plate scanner (Spectra Max 250; Molecular Devices, Menlo Park, CA, United States) at 490 nm. The results are expressed as picograms of TNF-α, IL-1β, and IL-6 per μL of the cell culture supernatant or serum. Optical density in the samples were derived from standard curves for each specific cytokine.

### Real-Time RT-PCR for TNF-α, IL-1β, and IL-6

Gene expression of TNF-α, IL-1β, and IL-6 was determined in mesenteric arteries and macrophages using the RNeasy kit (Qiagen Sciences). One micro gram of total RNA was reverse-transcribed in a final volume of 50 μL using the high-capacity cDNA archive kit (Applied Biosystems). TaqMan probes for TNF-α (Mm00443260_g1), IL-1β (Mm00434228_m1), IL-6 (Mm02620446_s1), and β-actin (Mm00607939_s1) mRNA were obtained from Applied Biosystems. Real-time RT-PCR (quantitative PCR) reactions were performed using the 7500 fast Real-Time PCR system (Applied Biosystems) in a total volume of 20 μL reaction mixture, following the manufacturer’s protocol, using the TaqMan fast universal PCR master mix (Applied Biosystems) and 0.1 mmol/L of each primer. Relative gene expression for TNF-α, IL-1β, and IL-6 mRNA were calculated with the 2^(–ΔΔCt)^ method and expressed as n-fold differences in TNF-α, IL-1β, and IL-6 gene expression relative to β-actin.

### Quantification of Nitric Oxide Production

The production of NO was indirectly determined by Griess reaction (Green et al., 1982) by measuring the content of nitrite (NO_2_^–^) in the cell culture supernatant. Fifty microliter of serum were incubated for 10 min (room temperature) in the presence of 50 μL Griess solution [1:1 (v/v) (solutions: sulfanilamide 0.1 mol/L ortho-phosphoric acid 30% and enediamine 7.7 mmol/L and 60% ortho-phosphoric acid]. Nitrite content was determined at 550 nm, and a curve of sodium nitrite (NaNO_2_) was used as standard.

### Blood Pressure Monitoring

To record arterial pressure, randomly selected mice from each experimental group were anesthetized with isoflurane (5% for induction and 2% 100% O_2_ for maintenance of anesthesia) for carotid artery catheterization (Intramedic, Becton Dickinson and Company). Three days after the surgical procedure, the catheter was connected to a transducer coupled to an amplifier system and to a computer (Dataquest LabPRO Acquisition System version 3.01, Data Sciences International, St. Paul, MN, United States). After a stabilization period, cardiovascular parameters (blood pressure and heart rate) were monitored for 6 h.

### Vascular Functional Studies

After euthanasia, the mesenteric beds from mice were rapidly removed and cleaned from fat tissue in an ice-cold (4°C) Krebs–Henseleit modified solution [(in mM): 130 NaCl, 4.7 KCl, 14.9 NaHCO_3_, 1.18 KH_2_PO_4_, 1.17 MgSO_4_⋅7H_2_O, 5.5 glucose, 1.56 CaCl_2_⋅2H_2_O, and 0.026 EDTA], gassed with 5% CO_2_/95% O_2_ to maintain a pH of 7.4. Second-order branches of mesenteric arteries (≈2 mm in length with internal diameter ≈150–200 μm) were carefully dissected and mounted as rings in an isometric Mulvany–Halpern myograph (model 610 M; Danish Myo Technology – DMT, Copenhagen, Denmark) (Halpern et al., 1978). Changes in force were recorded by a PowerLab 8/SP data acquisition system (ADInstruments). Second-order mesenteric arteries were adjusted to maintain a passive force of 13,3 kPa and allowed to equilibrate for about 30 min in Krebs–Henseleit solution. After the stabilization period, arterial integrity was assessed first by stimulation of vessels with 120 mM of KCl. After washing and a new stabilization period, endothelial function was assessed by testing the relaxant effect of acetylcholine (ACh, 10 μM) on vessels contracted with phenylephrine (PE, 2 μM). Mesenteric arteries exhibiting a relaxant response to ACh greater than 90% were considered endothelium-intact vessels. All experiments were performed in endothelium-intact vessels. Concentration-response curves to phenylephrine (PE; 1 nM to 100 μM) were performed. Endothelium-dependent relaxation was assessed by measuring the relaxation response to ACh (1 nM to 100 μM) in PE-contracted vessels (1 μM).

### Bone Marrow-Derived Macrophages

Bone marrow cells were harvested from femurs of control mice and differentiated in the presence of L929-conditioned medium (30%) in complete RPMI 1640 medium containing 10 mM glucose, 2 mM L-glutamine, 100 U/ml of penicillin/streptomycin and 20% fetal bovine serum (FBS). The culture medium was replaced on day 4. After 7 days, bone marrow-derived macrophages (BMDM) were counted and 2 × 10^6^ cells were incubated with glucosamine (GlcN, 5 mM, 30 min) or thiamet-G (ThG, 1 μM, 12 h) prior to 6 h stimulation with ultrapure LPS, 1 μM. Gene expression of cytokines was determined by Real-Time PCR.

### NF-κB Activity

To analyze whether *O*-GlcNAc modulates NF-κB activity, NF-κB luciferase stable RAW264.7 cells were used (Cooper et al., 2010). The murine leukaemic monocyte macrophage 264.7 cell line bearing the luciferase vector inserted in the NF-κB promoter (pNF-κB-Luc) was cultured in RPMI1640 medium supplemented with 10% FBS and antibiotics at 37°C in a humidified atmosphere of 5% CO_2_. For luciferase assay, 3 × 10^5^ cells were grown in 24 well-plates for 2 h. The cells were incubated with glucosamine (GlcN, 5 mM, 30 min), thiamet-G (ThG, 1 μM, 12 h) or vehicle, before stimulation with LPS (1 μM for 6 h). After LPS stimulation, cells were harvested and disrupted in lysis buffer (Tris–HCl 0.02 M, NaCl 0.08 M, TritonX-100 1%). The luciferase activity in the cell lysates was measured with the Luciferase Assay System (Promega, WI, United States), according to the manufacturer’s instructions using FlexStation^®^ 3 (Molecular Devices, United States).

### Western Blot Analysis

Aorta and mesenteric arteries were frozen in liquid nitrogen and homogenized in a lysis buffer [50 mM Tris/HCl, 150 mM NaCl, 1% Nonidet P40, 1 mM EDTA, 1 μg/ml leupeptin, 1 μg/ml pepstatin, 1 μg/ml aprotinin, 1 mM sodium orthovanadate, 1 mM phenylmethylsulfonyl fluoride (PMSF), and 1 mM sodium fluoride]. Proteins were extracted and separated (60 μg) by electrophoresis. Non-specific binding sites were blocked with 5% bovine serum albumin (BSA) in Tris-buffered saline (TBS) containing 0.1% Tween 20 (for 1 h at 24°C). Membranes were incubated with antibodies (at the indicated dilutions) at 4°C overnight. Antibodies were as follows: anti–*O*-GlcNAc antibody, CTD 110.6 (1:500; Sigma-Aldrich, O7764), phosphor-Ser^536^-p65 (cell signaling, #3033); NF-κB subunit p65 (cell signaling, #8242) and β-actin (1:2000; Sigma-Aldrich, A3854). After incubation with secondary antibodies, signals were obtained by chemiluminescence, and optical densitometry units were measured using Image Quant software (GE healthcare life sciences). Data are shown as the ratio of interest protein by β-actin band densitometry.

### Immunoprecipitation Assay

To verify whether NF-κB p65 subunit was modified by *O*-GlcNAc, NF-κB p65 subunit was immunoprecipitated, using magnetic beads (Millipore, LSKMAGG02) and anti-p65 subunit antibody (Cell Signaling #8242), from mesenteric arteries of control and LPS-induced SIRS mice treated with Thiamet-G or vehicle. *O*-GlcNAc levels were determined by western blot analysis using a monoclonal anti-β-*O*-Linked N-acetylglucosamine antibody (Sigma-Aldrich, O7764), as described above. Data are shown as the densitometric ratio of *O*-GlcNAc by NF-κB p65 subunit.

### Statistical Analysis

Prism software, version 5.0 (GraphPad Software Inc., San Diego, CA, United States) was used to analyze the parameters. Data are presented as mean ± SEM. Groups were compared using one-way ANOVA. ANOVA was followed by the Bonferroni’s or Dunnett’s post-test. Survival curves were analyzed with the log-rank test. *N* represents the number of animals used and *p* values less than 0.05 were considered significant.

## Results

### Effects of Increased *O*-GlcNAc on LPS-Induced Mice Mortality

**FIGURE 1:**
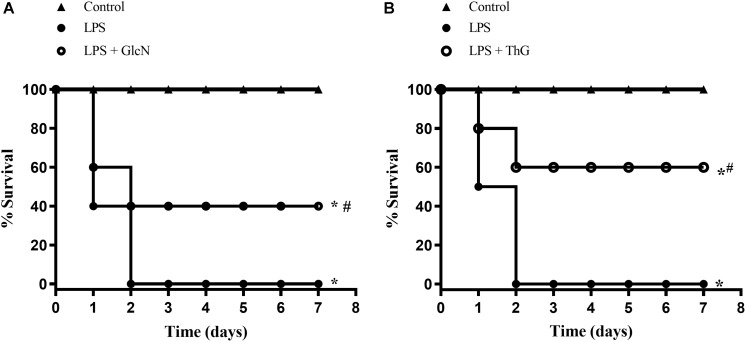
Survival rates in mice submitted to LPS-induced SIRS (20 mg/Kg, i.p.), and pre-treated with vehicle (LPS), **(A)** GlcN (300 mg/Kg, i.v., LPS + GlcN) or **(B)** ThG (150 μg/Kg, i.v., LPS + ThG). Survival rates were determined every 12 h for 7 days and the results are expressed as the percentage of animals alive on each day. ^∗^*p* < 0.05 vs. control. ^#^*p* < 0.05 vs. LPS. *N* = 10 animals per experimental group. Log-rank test.

To determine whether acute increases in *O*-GlcNAc levels affects survival rates in mice with LPS-induced SIRS, animals were followed for 7 days, and checked every 12 h. Mice undergoing LPS-induced SIRS (20 mg/Kg, i.p., LPS) died within 48 h. Treatment of LPS mice with GlcN, which increases the synthesis of UDP-GlcNAc in the hexosamine pathway, increased survival to 40%, when compared to mice that received only LPS (Figure 1A). Additionally, inhibition of the OGA enzyme with ThG improved survival of LPS-treated mice by up to 60% (Figure 1B).

### Effects of Increased *O*-GlcNAc on Inflammatory Response

#### Leukocyte Infiltration in Lungs and Leukocyte Migration to the Peritoneal Cavity

**FIGURE 2:**
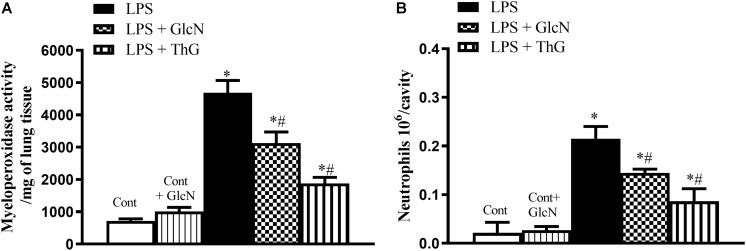
Neutrophil infiltration in the lung and neutrophil migration to the peritoneal cavity. Evaluation of **(A)** neutrophil infiltration in the lungs and **(B)** migration of neutrophils to the peritoneal cavity in mice submitted to LPS-induced SIRS and treated with vehicle (LPS), GlcN (LPS + GlcN) or ThG (LPS + ThG). Lung neutrophil sequestration was estimated by the myeloperoxidase assay (MPO) in the organ homogenate 6 h after LPS-induced SIRS. Neutrophil count into the peritoneal cavity was performed using the Coulter^®^ apparatus. The results are expressed as mean ± SEM and are representative of 4–5 experiments. ^∗^*p* < 0.05 vs. control; ^#^*p* < 0.05 vs. LPS. One-way ANOVA followed by Dunnett’s post-test.

Sequestration of neutrophils from the circulation is an event that can compromise an appropriate response to infection (Sumi et al., 2014). To evaluate the effect of increased *O*-GlcNAc on neutrophil infiltration in the lungs, myeloperoxidase (MPO) activity was determined. Figure 2A demonstrates that treatment with both GlcN and ThG significantly reduced neutrophil sequestration in the lung of LPS mice as compared to vehicle-treated mice. In addition to reducing pulmonary neutrophil sequestration, increased *O*-GlcNAc also decreased neutrophil migration to the peritoneal cavity (Figure 2B).

#### Systemic Levels and Vascular Expression of Inflammatory Cytokines

**FIGURE 3:**
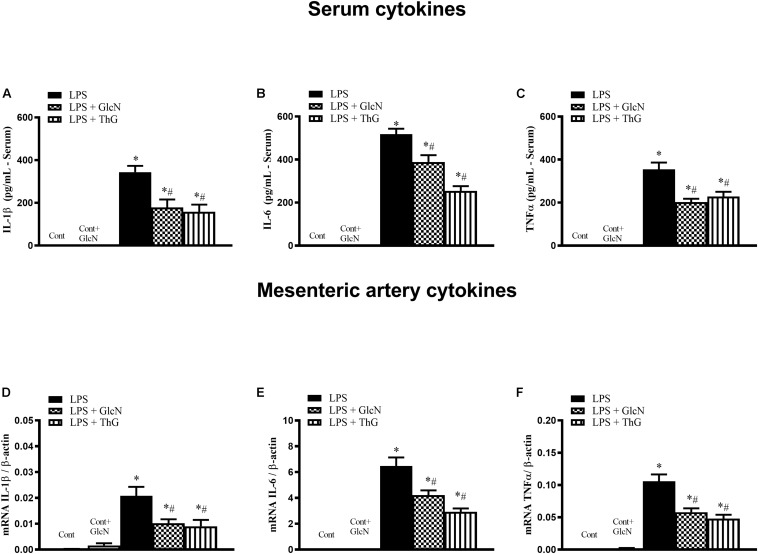
Systemic and vascular production of IL-1β, IL-6, and TNF-α. Evaluation of **(A–C)** serum concentration and **(D–F)** mesenteric artery mRNA of **(A,D)** IL-1β, **(B,E)** IL-6, and **(C,F)** TNF-α in mice submitted to LPS-induced SIRS and treated with vehicle (LPS), GlcN (LPS + GlcN) or ThG (LPS + ThG). The results are expressed as mean ± SEM and are representative of 3–4 experiments. ^∗^*p* < 0.05 vs. control; ^#^*p* < 0.05 vs. LPS. One-way ANOVA followed by Dunnett’s post-test.

The production of inflammatory cytokines coordinates the response to infectious agents through the activation and recruitment of immune cells (Dinarello, 1997; Alves-Filho et al., 2010; Li et al., 2019). To determine the production of inflammatory mediators, serum concentrations of IL-1β, IL-6, and TNFα were quantified. mRNA expression of these cytokines was determined in mesenteric arteries. LPS increased both serum levels and vascular expression of IL-1β, IL-6 and TNFα (Figure 3). Treatment of LPS-mice with GlcN and ThG attenuated systemic levels (Figures 3A,C) and vascular expression (Figures 3D,F) levels of cytokines. These results suggest that increased *O*-GlcNAc reduces LPS-associated pro-inflammatory cytokines production.

To confirm that acute increases in *O*-GlcNAc protein levels has anti-inflammatory effects, we also used a lower dose of LPS (LPS_low_) to induce SIRS. Accordingly, experiments were performed in mice injected via the intraperitoneal route with 10 mg/Kg LPS.

Supplementary Figure S2 shows that treatment of LPS_low_ mice with GlcN and ThG decreased systemic levels of IL-1β, IL-6 and TNFα, indicating that acute increase in *O*-GlcNAc reduces pro-inflammatory events in mice subjected to both low and high doses of LPS.

### Effect of Increased *O*-GlcNAc Levels in the Cardiovascular System

#### LPS-Induced Hypotension

**FIGURE 4:**
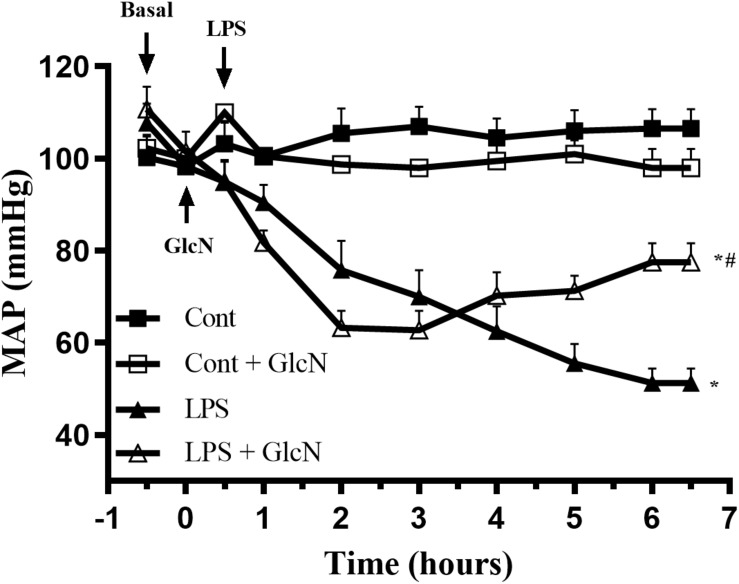
Mean arterial pressure (MAP) in LPS-induced SIRS in mice. Mean arterial pressure (MAP) was recorded, via carotid artery cannulation, for 6 h after LPS administration (20 mg/kg, i.p.). GlcN treatment was performed 30 min before LPS injection. The results are expressed as mean ± SEM and are representative of four experiments. ^∗^*p* < 0.05 vs. control; ^#^*p* < 0.05 vs. LPS. One-way ANOVA followed by Bonferroni’s post-test.

Hypotension contributes to organ failure in SIRS-associated conditions (De Backer et al., 2014; Coeckelenbergh et al., 2019). LPS induces a progressive decrease in mean arterial blood pressure (MAP) in mice. Although GlcN did not prevent LPS-induced hypotension, the fall of MAP was attenuated 5 h after LPS injection (Figure 4).

Mice that received a moderate dose of LPS (10 mg/Kg i.p.) also exhibited hypotension, as shown in Supplementary Figure S3. Similarly, GlcN treatment did not prevent hypotension in mice with LPS_low_ (LPS_low_ + GlcN), but MAP was significantly higher 5 h after LPS-induced endotoxemia (Supplementary Figure S3).

#### Vascular Reactivity

**FIGURE 5:**
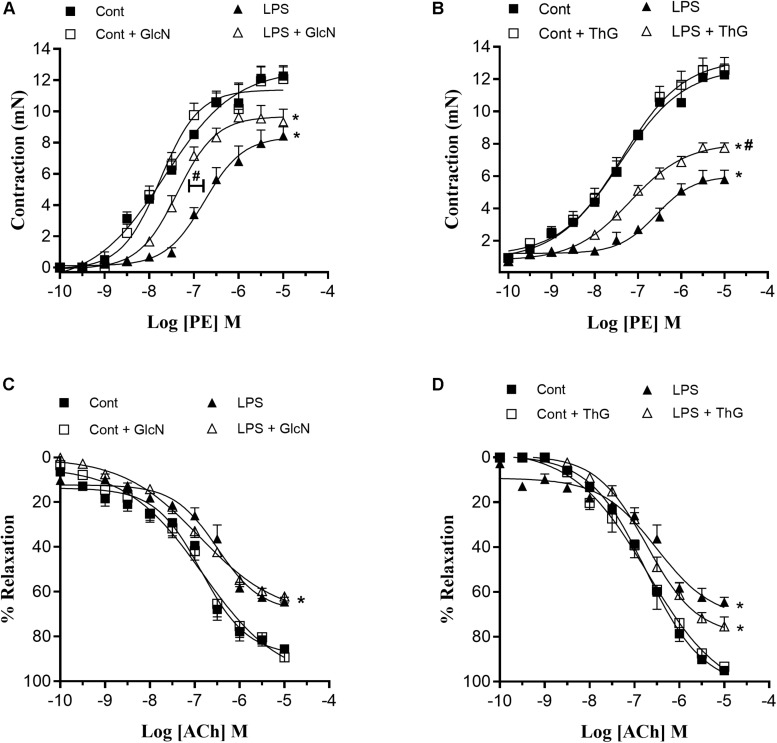
Mesenteric artery reactivity. **(A,B)** Contractile responses to phenylephrine (PE) and **(C,D)** relaxation responses to acetylcholine (ACh) in mesenteric arteries from control and LPS-induced SIRS (LPS) mice treated with **(A,C)** vehicle or glucosamine (GlcN) and **(B,D)** vehicle or thiamet-G (ThG). Data are expressed as mean ± SEM of contraction and relaxation values and are representative of 4–5 experiments. Cont, vehicle-treated control mice; LPS, LPS (20 mg/Kg i.P.)-treated mice; Cont + GlcN and LPS + GlcN, mice treated with glucosamine (GlcN); Cont + ThG and LPS + ThG, mice treated with thiamet-G (ThG). ^∗^*p* < 0.05 vs. respective control; ^#^*p* < 0.05 vs. LPS. ANOVA followed by Bonferroni’s post-test.

In sepsis, hyporesponsiveness to vasopressor agents contributes to the reduction of MAP and organ perfusion (De Backer et al., 2014; Ozer et al., 2017). Therefore, vascular function was determined by evaluating mesenteric artery responses to phenylephrine (PE) and acetylcholine (ACh). LPS-induced SIRS reduced mesenteric artery reactivity to phenylephrine and reduced vasodilator responses to ACh (Figures 5A,C). Moreover, the concentration-response curves to phenylephrine in mesenteric arteries from LPS mice treated with GlcN and ThG showed a shift to the left, indicating improvement of contractile vascular responses (Figures 5A,B and Tables 1, 2). Treatment with GlcN and ThG did not restore maximal contractile responses (Emax) to phenylephrine (Figures 5A,B) or the reduced vasodilation to ACh induced by LPS (Figures 5C,D and Table 1).

**TABLE 1:** Effect of GlcN treatment on Emax and pD_2_ values for phenylephrine and acetylcholine in mesenteric arteries from mice submitted to LPS-induced SIRS and treated with vehicle or GlcN.

	PE		ACh	
	Emax	pD_2_	Emax	pD_2_
Cont	12.69 ± 0.73	7.65 ± 0.14	87.18 ± 2.89	7.65 ± 0.08
Cont + GlcN	11.75 ± 0.54	7.76 ± 0.12	95.04 ± 4.51	7.39 ± 0.11
LPS	8.40 ± 0.49^∗^	6.76 ± 0.10^∗^	66.70 ± 1.40^∗^	7.03 ± 0.08^∗^
LPS + GlcN	9.53 ± 0.30^∗^	7.37 ± 0.07^*,#^	66.56 ± 2.89^∗^	7.48 ± 0.10^∗^

*Emax and pD_2_ values for phenylephrine (PE) and acetylcholine (ACh) in mesenteric arteries from mice of the following groups: control (Cont), control + GlcN treatment (cont + GlcN), LPS (LPS), and LPS + GlcN treatment (LPS + GlcN). Data are expressed as mean ± SEM of contraction and relaxation values and are representative of 4–5 experiments. ^∗^*p* < 0.05 vs. control group; ^#^*p* < 0.05 vs. LPS group. ANOVA followed by Bonferroni’s post-test.*

**TABLE 2:** Effect of ThG treatment on Emax and pD_2_ values for phenylephrine (PE) and acetylcholine (ACh) in mesenteric arteries from mice submitted to LPS-induced SIRS and treated with thiamet-G.

	PE		ACh	
	Emax	pD_2_	Emax	pD_2_
Cont	12.72 ± 0.73	7.43 ± 0.14	95.05 ± 0.71	6.73 ± 0.05
Cont + ThG	13.24 ± 0.68	7.36 ± 0.12	93.30 ± 1.14	6.70 ± 0.18
LPS	6.30 ± 0.57^∗^	6.54 ± 0.18^∗^	64.54 ± 2.07^∗^	6.50 ± 0.16^∗^
LPS + ThG	8.05 ± 0.28^∗^	7.19 ± 0.08^*,#^	75.36 ± 4.18^∗^	6.72 ± 0.80^∗^

*Emax and pD_2_ values for PE contraction and ACh relaxation in mesenteric arteries from mice of the following groups: control (Cont), control treated with ThG (cont + ThG), treated with LPS (LPS), and LPS + ThG treatment (LPS + ThG). Data are expressed as mean ± SEM of contraction and relaxation values and are representative of 4–5 experiments. ^∗^*p* < 0.05 vs. control; ^#^*p* < 0.05 vs. LPS. ANOVA followed by Bonferroni’s post-test.*

Similarly, OGA inhibition by treatment with ThG improved mesenteric artery contractile responses to phenylephrine (Figure 5B and Table 2), but it did not change the decreased relaxation responses to ACh in LPS mice (Figure 5D and Table 2).

Similar responses were observed in mesenteric arteries from mice treated with the lower dose of LPS (10 mg/kg i.p.), i.e., responses to phenylephrine were decreased in LPS_low_ mice. Arteries from LPS_low_ mice treated with ThG showed increased responses to phenylephrine, indicating improvement of contractile vascular responses (Supplementary Figure S4).

### Nitrite Quantification and Inducible Nitric Oxide Synthase Expression

**FIGURE 6:**
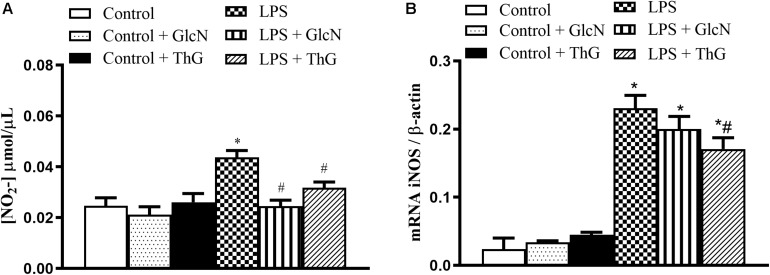
Nitrite levels and vascular iNOS mRNA expression. **(A)** Glucosamine (GlcN) and Thiamet G (ThG) treatment prevented the increase in systemic nitrite levels in LPS mice. Serum was collected 6 h after induction of SIRS (LPS). Nitrite production was determined by the Griess method. **(B)** ThG treatment attenuated inducible nitric oxide synthase (iNOS) gene expression in mesenteric arteries from mice with SIRS (2^–ΔΔ*CT*^). Results are expressed as mean ± SEM and are representative of 3–4 experiments. ^∗^*p* < 0.05 vs. control; ^#^*p* < 0.05 vs. LPS. ANOVA followed by Dunnett’s multiple comparisons test.

Vascular hyporesponsiveness in LPS-induced SIRS has been attributed to an excessive production of vasodilator mediators, such as nitric oxide (NO) (Kandasamy et al., 2011). To determine NO production, its secondary metabolite (nitrite) was measured. LPS mice showed a significant increase in nitrite serum levels, which was prevented by GlcN and ThG treatments (Figure 6A). In addition, OGA inhibition with ThG, but not GlcN treatment, attenuated the increased iNOS mRNA vascular expression induced by LPS (Figure 6B).

### Cytokine Production by Macrophages

**FIGURE 7:**
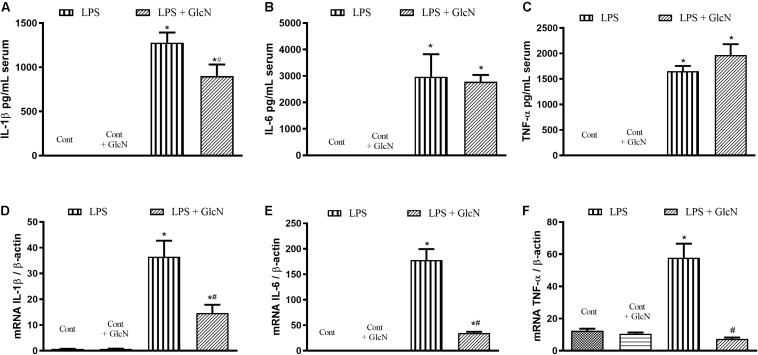
LPS-induced pro inflammatory profile of bone marrow-derived macrophages (BMDM) treated with vehicle or glucosamine. BMDM were isolated from control mice. BMDM were treated with glucosamine (GlcN, 1 μM, 30 min) and then with LPS (1 μM for 6 h). The supernatant was collected to measure IL-1β **(A)**, IL-6 **(B)**, and TNFα **(C)** cytokines by ELISA. Macrophages mRNA levels of IL-1β **(D)**, IL-6 **(E)**, and TNFα **(F)** were determined by qPCR. Data are represented by mean ± SEM and are representative of 3–5 experiments. ^∗^*p* < 0.05 vs. control; ^#^*p* < 0.05 vs. LPS. ANOVA followed by Dunnett’s multiple comparisons test.

Acute increases in *O*-GlcNAc proteins reduced LPS-associated inflammatory processes in mice, as shown in Figure 3. Considering that macrophages are important cells in the response to infectious agents, LPS-induced pro-inflammatory response was determined in bone marrow-derived macrophages (BMDM). BMDM were isolated from control naïve mice and treated *in vitro* with LPS (1 μM, 6 h). LPS-treated BMDM showed increased secretion of pro-inflammatory cytokines (Figures 7A–C) as well as increased mRNA expression of IL-1β (Figure 7D), IL-6 (Figure 7E), and TNFα (Figure 7F). GlcN treatment reduced LPS-induced IL-1β secretion in BMDM (Figure 7A). Also, GlcN treatment decreased mRNA levels of IL-1β (Figure 7D), IL-6 (Figure 7E), and TNFα (Figure 7F) in BMDM stimulated with LPS.

**FIGURE 8:**
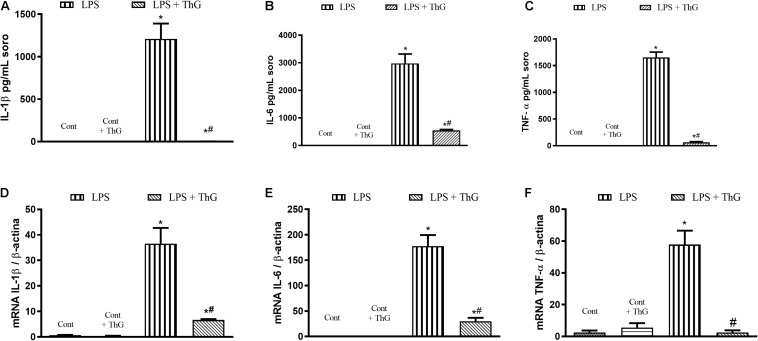
Pro inflammatory profile of bone marrow-derived macrophages (BMDM) from control mice stimulated with LPS in the presence of vehicle or thiamet G. BMDM were isolated from control mice. BMDM were treated with thiamet G (ThG, 1 μM, 12 h) and then, with LPS (1 μM, 6 h). The supernatant was collected to measure cytokines IL-1β **(A)**, IL-6 **(B)**, and TNFα **(C)** by ELISA. The cells were lysed to evaluate mRNA levels of IL-1β **(D)**, IL-6 **(E)**, and TNFα **(F)** by qPCR. Data are represented by mean ± SEM and are representative of 3–5 experiments. ^∗^*p* < 0.05 vs. control; ^#^*p* < 0.05 vs. LPS. ANOVA followed by Dunnett’s multiple comparisons test.

Moreover, OGA inhibition with ThG also decreased LPS-induced pro-inflammatory responses in BMDM, reducing cytokines secretion and mRNA expression of IL-1β (Figures 8A,D), IL-6 (Figures 8B,E), and TNFα (Figures 8C,F).

### NF-κB Activity

**FIGURE 9:**
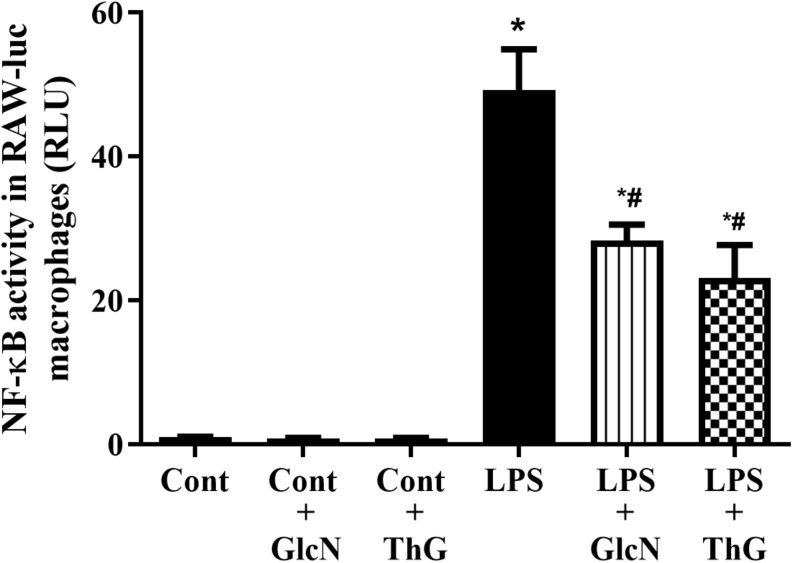
NF-κB activation in macrophages. Glucosamine (GlcN, 5 mM, 30 min) and thiamet G (ThG, 1 μM 12 h) attenuated LPS-induced NF-κB activation in RAW 264.7 macrophages. NF-κB activity was measured after incubation with LPS (1 μM for 6 h). Data are represented as mean ± SEM of RLU (relative luminescence unit) and are representative of 4–8 experiments. ^∗^*p* < 0.05 vs. control; ^#^*p* < 0.05 vs. LPS. ANOVA followed by Dunnett’s multiple comparisons test.

Considering that acute increases in *O*-GlcNAc proteins reduced cytokines production and that NF-κB is a key transcription factor that induces IL-1β and TNF-α mRNA expression, we determined whether acute increase in *O*-GlcNAc proteins reduces NF-κB signaling. As shown in Figure 9, treatment of RAW 264.7 NF-κB promoter macrophages with GlcN or ThG reduced LPS-induced NF-κB activation.

### *O*-GlcNAc Modification of NF-κB p65 Subunit in Mesenteric Arteries

#### Expression of Total, Phosphorylated and *O*-GlcNAc-Modified NF-κB p65 Subunit

**FIGURE 10:**
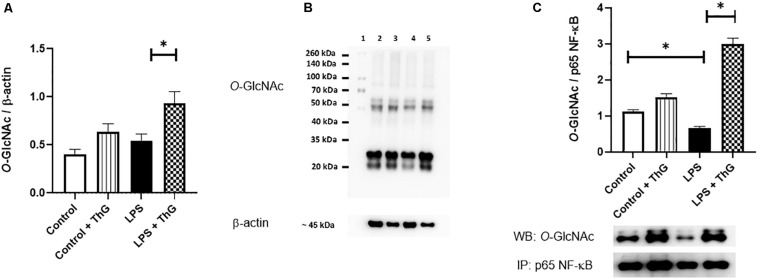
*O*-GlcNAc protein levels in mesenteric arteries from Control and LPS mice treated with vehicle or Thiamet-G. **(A)** Global quantification and **(B)** representative images of *O*-GlcNAc protein levels in mesenteric arteries from the experimental groups. Lanes: (1) molecular standard protein weight; (2) control group with saline; (3) control group treated with Thiamet-G (ThG, 1 μM 12 h); (4) LPS-induced SIRS group treated with saline; (5) LPS-induced SIRS group treated with ThG. **(C)** *O*-GlcNAcylated NF-κB p65 subunit, determined by immunoprecipitation, in mesenteric arteries from control and LPS mice. Data are represented as mean ± SEM ^∗^*p* < 0.05, *N* = 3–5 different experiments. One-way ANOVA followed by Tukey’s multiple comparisons test.

**FIGURE 11:**
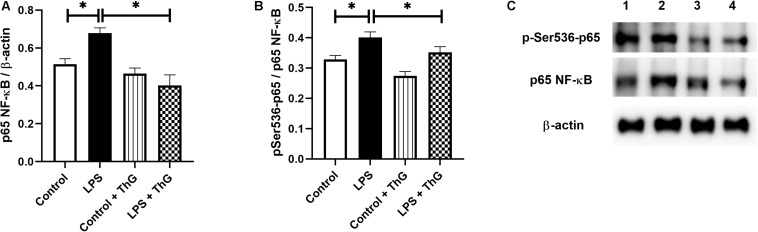
Expression of total and phosphorylated NF-κB p65 subunit. Graphs show expression of **(A)** total and **(B)** phosphorylated (at the residue serine^536^) forms of the p65 subunit. **(C)** Representative image of 4 different experiments. Lanes in C: (1) control group with saline, (2) LPS-induced SIRS group treated with saline, (3) control group treated with Thiamet-G (1 μM, 12 h) and (4) LPS-induced SIRS group treated with Thiamet-G (1 μM, 12 h). Data are represented as mean ± SEM. ^∗^*p* < 0.05. One-way ANOVA followed by Tukey’s multiple comparisons test.

Since activation of the hexosamine pathway decreased inflammatory responses and improved vascular function in mice with LPS-induced SIRS, *O*-GlcNAc protein levels in mesenteric arteries were quantified by western blot analysis. ThG increased *O*-GlcNAc levels in mesenteric arteries from control (*p* = 0.06) and LPS-treated mice (*p* < 0.05) (Figures 10A,B). LPS-treated mice showed a decrease in *O*-GlcNAc-modified NF-κB p65 subunit, which was not observed in mice treated with ThG (Figure 10C). Mesenteric arteries from mice with LPS-induced SIRS showed increased expression of the total (Figure 11A) and phosphorylated (Figure 11B) forms of NF-κB p65 subunit, which was not observed in mice treated with ThG (Figure 11C).

### Cecal Ligation and Puncture (CLP) Sepsis Model in Mice

#### Effect of GlcN on CLP-Induced Mortality

**FIGURE 12:**
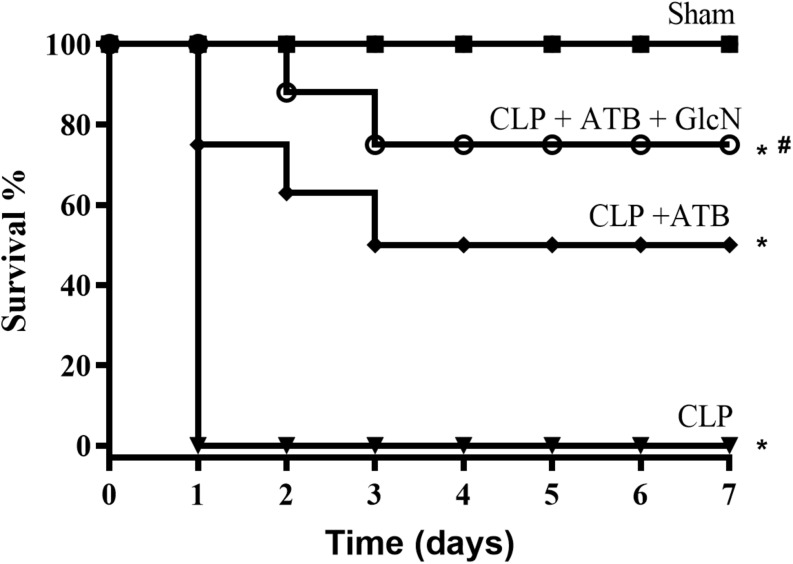
Survival rate in mice of the control group (Sham) and mice submitted to cecal ligation and puncture-induced sepsis (CLP); CLP + ATB (treatment with sodium ertapenem 30 mg/kg s.c.), and CLP + ATB + GlcN (300 mg/Kg, i.v.). Survival rates were determined every 12 h for 7 days and the results are expressed as the percentage of animals alive on each day. ^∗^*p* < 0.05 vs. control. ^#^*p* < 0.05 vs. CLP + ATB. *N* = 10 animals per experimental group. Log-rank test.

To evaluate the effects of increased *O*-GlcNAc proteins in a second experimental model, GlcN was administrated to mice with sepsis induced by CLP. CLP-induced sepsis was lethal in 24 h. Treatment of mice with hydration plus antibiotic (Ertapenem, 30 mg/Kg) every 12 h for three consecutive days reduced mortality by approximately 50%. Moreover, GlcN improved the hydration + antibiotic effect, resulting in survival rates of 80% (Figure 12).

#### Leukocyte Infiltration in Lungs and Leukocyte Migration to the Peritoneal Cavity in CLP Treated Mice

**FIGURE 13:**
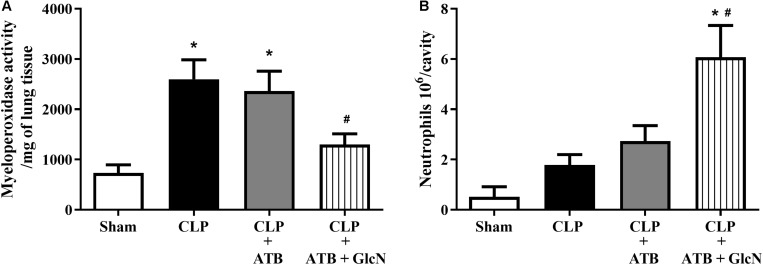
Neutrophil infiltration in the lung and neutrophil migration to the peritoneal cavity in CLP mice. Evaluation of **(A)** neutrophil infiltration in the lungs and **(B)** migration of neutrophils to the peritoneal cavity in control mice (Sham) and mice submitted to CLP-induced sepsis; CLP mice treated with sodium ertapenem (30 mg/kg s.c., CLP + ATB), or CLP + ATB + GlcN (300 mg/Kg, i.v., CLP + ATB + GlcN). Lung neutrophil sequestration was estimated by the myeloperoxidase assay (MPO) in the organ homogenate 6 h after CLP-induced sepsis. Neutrophil count into the peritoneal cavity was performed using the Coulter^®^ apparatus. The results are expressed as mean ± SEM and are representative of 4–5 experiments. ^∗^*p* < 0.05 vs. sham; ^#^*p* < 0.05 vs. CLP + ATB. One-way ANOVA followed by Dunnett’s post-test.

Treatment with GlcN significantly reduced neutrophil sequestration in the lung in mice with CLP-induced sepsis as compared to Sham mice (Figure 13A). GlcN treatment increased neutrophil migration to the peritoneal cavity (Figure 13B).

## Discussion

Early events in an inflammatory process involve the activation of immune cells such as polymorphonuclear leukocytes (neutrophils), monocytes/macrophages and lymphocytes. Activation of immune cells leads to production and secretion of proinflammatory mediators, which are responsible for most of the pathophysiological changes in endotoxemia/sepsis (Dinarello, 1997; De Backer et al., 2014; Li et al., 2019). Here, we show that acute increase in *O*-glycosylation with N-acetylglucosamine (*O*-GlcNAc) reduces inflammatory processes and improves cardiovascular function in mice with moderated and severe endotoxemia. More specifically, increased *O*-GlcNAc attenuates hypotension, improves contractile vascular responses and reduces systemic and local proinflammatory cytokine production by mechanisms that involve reduced NF-κB activity.

In conditions of systemic inflammatory responses, such as sepsis, exacerbated inflammation contributes to multiple organ dysfunction, that is strictly related to loss of vasomotor tone, decreased peripheral vascular resistance, decreased blood pressure and cardiac output, hypoperfusion, and hypoxygenation of tissues and organs leading to a significant mortality in intensive care units (De Backer et al., 2014; Hotchkiss et al., 2016; Machado et al., 2017; Salomão et al., 2019). This study shows that acute protein glycosylation, either by increasing flux in the hexosamine pathway, with glucosamine, or by OGA inhibition, with thiamet-G, improves survival of mice with severe endotoxemia in 40 and 60% (Figure 1), respectively, suggesting that acute increase in *O*-GlcNAc reduces mortality associated to septic condition. Furthermore, in the CLP-induced sepsis model, that is an important model of sepsis (Alves-Filho et al., 2010), GlcN treatment potentialized the antibiotic effect in improving septic animal survival (Figure 12).

The disarrangement in the immune response in the septic patient is an important risk factor that leads to tissue injury and organ dysfunction. This process involves the activation of components of the innate immune response, including humoral and cellular components. An efficient immune response is critical for local control and inhibition of the inflammatory process in LPS-induced SIRS (Cohen, 2002) and neutrophil migration is key in the pathogenesis of endotoxemia (Alves-Filho et al., 2010). Accordingly, increased pulmonary neutrophil sequestration, increased neutrophil numbers in abdominal cavity and high levels of systemic IL-1β, IL-6, and TNFα (Figures 2, 3, 13 and Supplementary Figures S2, S5) were observed in LPS-treated and CLP-induced septic mice, depicting a disruption and uncontrolled activation of the immune system in septic mice. Moreover, GlcN and ThG attenuated lung neutrophil sequestration and the number of neutrophils in the abdominal cavity (Figures 2, 13). The reduction in lung neutrophil accumulation can related a lower lung injury and contribute to improvement in animal survival. In CLP model, GlcN also reduce the lung neutrophils sequestration (Figure 13A). Moreover, in this septic model, increase in O-GlcNAc protein levels result in higher neutrophils migration to peritoneal cavity (Figure 13B). This result could suggest that GlcN improve the neutrophils response to migrate to the focus of the infection. Although the mechanisms involved in neutrophil migration are not fully understood, it is known that high levels of circulating cytokines/chemokines (including TNF-α and IL-1β) modulate the failure of neutrophil migration during SIRS, by releasing nitric oxide (NO), mainly produced by NO inducible synthase (iNOS) (Farias Benjamim et al., 2002; Mestriner et al., 2007).

It is important to highlight that the treatment of LPS mice with GlcN and ThG prevented LPS-induced increase in nitrite serum concentration and ThG also reduced iNOS mRNA in mice (Figure 6). Additionally, GlcN- and ThG-treated animals also showed lower serum concentration of IL-1β, TNFα, and IL-6 (Figure 3 and Supplementary Figure S5). These *O*-GlcNAc-induced modifications potentially explain the reduction of lung neutrophil sequestration in this study.

GlcN has anti-inflammatory properties in various animal models and cell types (Shikhman et al., 2001; Gouze et al., 2002; Largo et al., 2003; Chen et al., 2006; Zou et al., 2009; Shin et al., 2013). GlcN treatment reduces the expression of inflammatory mediators, such as IL-6 and cyclooxygenase 2 (COX2) in human chondrocytes (Shikhman et al., 2001; Largo et al., 2003), inhibits IL-1β activity in rat chondrocytes (Gouze et al., 2002), decreases TNF-α-induced ICAM expression in human conjunctival epithelial cells (Chen et al., 2006) as well as LPS-induced NO production in macrophage cell lines (Shin et al., 2013). These studies corroborate the hypothesis that increased *O*-GlcNAc-modified proteins reduce levels of circulating pro-inflammatory cytokines with potential protective effects in septic conditions, as observed in our study.

In septic conditions, decreased arterial blood pressure, arterial hyporesponsiveness to contractile agents and vasoplegia are the main symptoms that lead to diminished circulatory blood volume and poor septic patient prognosis (Kandasamy et al., 2011; De Backer et al., 2014; Sumi et al., 2014; Ozer et al., 2017; Coeckelenbergh et al., 2019). For this reason, restoration of vascular function is key in sepsis therapy. In this study, we observed that GlcN treatment attenuated arterial hypotension induced by LPS (Figure 4) and increased mesenteric artery sensitivity to phenylephrine (Figure 5). OGA inhibition also improved mesenteric artery contractile response to phenylephrine in moderated and severe LPS-induced SIRS (Figure 5 and Supplementary Figure S4). Taken together, these results suggest that increased *O*-GlcNAc protein levels have positive effects in septic condition, improving MAP, vascular function and, consequently, animal survival.

Excessive inflammatory response promotes vascular damage, intravascular systemic vasodilation, hyporesponsiveness to vasopressors, multiple organ failure, and mortality. In our study, SIRS significantly increased cytokines circulating levels, which can contribute to vascular dysfunction in septic animals. Previous studies have shown that pro-inflammatory cytokines, such as IL-1β and TNFα, activate vascular cells to produce more proinflammatory cytokines, which exacerbates the inflammatory response and stimulates generation of vasoactive molecules, such as NO. For instance, Xing et al. (2011) showed that TNF-α induces iNOS expression through the NF-κB signaling pathway in aortic smooth muscle cells and Dalvi et al. (2017) observed that activated monocyte exosomes stimulate IL-1β and IL-6 production by endothelial cells. Corroborating these studies, we observed an increase in mesenteric artery mRNA levels of IL-1β, TNFα, and IL-6 (Figure 3), which was paralleled by increased expression of the total and phosphorylated forms of the NF-κB p65 subunit (Figure 11), suggesting a local proinflammatory vascular response in septic animals by activation of NF-κB signaling. Activation of the hexosamine pathway by GlcN and OGA inhibition by ThG prevented the systemic and local production of pro-inflammatory cytokines. Furthermore, treatment of mice with ThG increased *O*-glycosylation of the NF-κB p65 subunit in mesenteric arteries, which was associated with reduced Ser^536^ phosphorylation of the p65 subunit (Figures 10, 11, respectively). NF-κB p65 subunit phosphorylation is key to NF-κB-dependent TNFα production (Ahmed et al., 2014) and decreased phosphorylation of NF-κB p65 may explain the anti-inflammatory effect of ThG treatment. All these effects may synergistically contribute to ThG-mediated improved vascular function in LPS-treated animals.

Macrophages have a crucial role in septic inflammatory response. During infection, activated macrophages increase the production of inflammatory mediators to fight the infectious agent and to recruit other immune cells. However, a massive synthesis of proinflammatory mediators contributes to all sepsis-related deleterious effects, as discussed above (Cohen, 2002; van der Poll et al., 2017; Salomão et al., 2019). In our study, BMDM isolated from animals produced the highest levels of cytokines when they were challenged with LPS (Figures 7, 8). Furthermore, *in vitro* experiments, GlcN and ThG treatments attenuated the production and secretion of IL-1β, TNFα, and IL-6 by BMDM (Figures 7, 8). These results suggest that the positive effects induced by GlcN and ThG treatment in septic animals may result from reduced macrophages response.

A recent study showed that LPS-activated macrophages show lower levels of *O*-GlcNAc proteins and activation of OGT-mediated *O*-GlcNAcylation reduces pro-inflammatory macrophages response by inhibition of receptor-interacting serine/threonine-protein kinase 3 (RIPK3), which regulates diverse intracellular signaling, including NF-κB (Li et al., 2019). However, the cardiovascular impact of increased *O*-GlcNAc protein levels in the septic mice were not investigated. The inhibition of RIPK3 may help to explain how GlcN and ThG treatments improve cardiovascular function in septic mice. Additionally, we also observed that acute increase in *O*-GlcNAc proteins attenuates LPS-induced NF-κB activation in macrophages (Figure 9), indicating that modulation of NF-κB signaling in macrophages is important for the positive effect induced by *O*-GlcNAcylation in septic mice.

## Conclusion

In conclusion, our result provides strong evidence that acute increase of *O*-GlcNAc protein levels increases survival by reducing systemic inflammation and vascular dysfunction, suggesting that reprogramming of cellular metabolism, by stimulating the hexosamine pathway, may represent a therapeutic approach in septic conditions.

## Data Availability Statement

The datasets generated for this study are available on request to the corresponding author.

## Ethics Statement

The animal study was reviewed and approved by Ethics Committee on Animal Research of the Ribeirão Preto Medical School, University of São Paulo (protocol no 196/13) and are in accordance with the Guidelines of the National Council for Animal Experimentation Control (CONCEA).

## Author Contributions

VO, JA-F, and RT participated in the design of the study. JS, VO, CZ, RGF, NF, CS, JA, JL, and FM conducted the experiments. FC, JA-F, RF, and RT contributed with reagents or analytical tools. JS, VO, CZ, and RT performed the data analysis. JS, VO, and RT wrote the manuscript. All authors discussed and reviewed the manuscript.

## Conflict of Interest

The authors declare that the research was conducted in the absence of any commercial or financial relationships that could be construed as a potential conflict of interest.
