# Supplementary material for: Acute Increase in O-GlcNAc Improves Survival in Mice With LPS-Induced Systemic Inflammatory Response Syndrome
Source: Front Physiol. 2020 Jan 21;10:1614. doi: 10.3389/fphys.2019.01614 (PMC6985589; doi:10.3389/fphys.2019.01614)
Supplement: Supplementary file 1 [file Data_Sheet_1.docx]

**Supplementary Material**

**Acute increase in *O*-GlcNAc improves SURVIVAL in mice with LPS-induced systemic inflammatory response syndrome.**

Silva JF^1*^, Olivon VC^2*^, Mestriner FLAC^1,4^, Zanotto CZ^1^, Ferreira RG^1^, Ferreira NS^1^, da Silva CAA^3^, Luiz JPM^1^, Alves JV^1^, Fazan R^3^, Cunha FQ^1^, Alves-Filho JC^1^, Tostes RC^1^.

Departments of ^1^Pharmacology, ^3^Physiology and ^4^Surgery and Anatomy, Ribeirao Preto Medical School, University of Sao Paulo, Ribeirao Preto – SP, Brazil.

^2^Universidade Anhanguera-UNIDERP Campo Grande – MS, Brazil.

**Supplementary Material**

To increase *O*-GlcNAc-modified proteins in C57BL/6 mice, animals were treated with either glucosamine (GlcN, 300 mg/Kg i.v.) or thiamet G (ThG, 150 μg/Kg i.v.) before the induction of endotoxemia with a single dose (20 mg/Kg, i.p.) of LPS (Escherichia coli 0111:B4, Sigma Chemical Co. St. Louis, MO, USA). To determine the time with highest *O*-GlcNAc-modified protein levels, animals were sacrificed 0, 30 min, 1, 6 ,12 and 24 h after the administration of either GlcN or ThG and levels of *O*-GlcNAc-modified proteins in the aortae were used as an indication of global *O*-glycosylation.

**Supplementary Data**

Treatment with GlcN increased O-GlcNAc-modified proteins levels after 30 min of administration (***Supplementary Figures 1A and 1B)***, whereas inhibition of OGA with ThG significantly increased proteins modification by *O*-GlcNAc at 12 h (***Supplementary Figure 1C and 1D).***

***
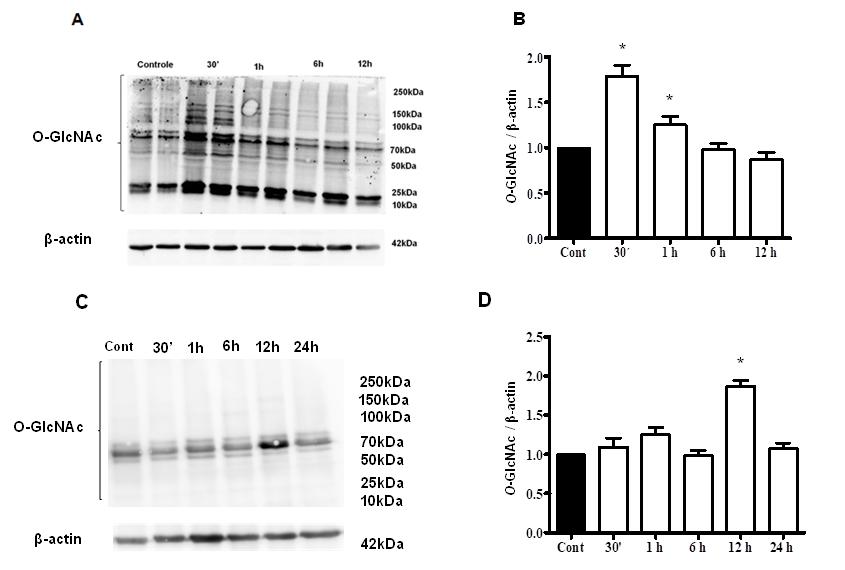
***

***Supplementary Figure 1*:** Glucosamine (GlcN, 300 mg/Kg i.v.) and Thiamet-G (ThG, 150 μg/Kg, i.v.) increase the content of *O*-GlcNAc-modified proteins in mice aorta. **A** - representative western blot image of *O*-GlcNAc-proteins in mice aorta, 30 min, 1, 6 and 12 h after treatment with GlcN. **B** - corresponding bar graphs showing relative expression of *O*-GlcNAc-modified proteins after normalization by β-actin. **C** - representative western blot image of *O*-GlcNAc-modified proteins in mice aorta, 30 min, 1, 6, 12 and 24 h after treatment with ThG. **D** - corresponding bar graphs showing the relative expression of *O*-GlcNAc-modified proteins after normalization to β-actin. Data are represented as mean ± SEM for *N*= 5 in each experimental group. *, *P*< 0.05 vs. control. ANOVA followed by Dunnett's multiple comparisons test).

**Moderate LPS-induced SIRS**

To determine whether acute increases in *O*-GlcNAc protein levels have anti-inflammatory effects also in moderate sepsis, experiments were performed in mice treated with a lower dose of lipopolysaccharide (LPS_low_, 10 mg/Kg, i.p.).

Similar to the treatment with high dose of LPS, LPS_low_ increased serum levels of IL-1β, IL-6 and TNFα (Supplementary Figure 2). Treatment of LPS_low_ mice with GlcN and ThG also decreased systemic levels of IL-1β, IL-6 and TNFα. Together, these results suggest that acute increased *O*-GlcNAc also reduces pro-inflammatory cytokines production in moderate LPS-induced SIRS.


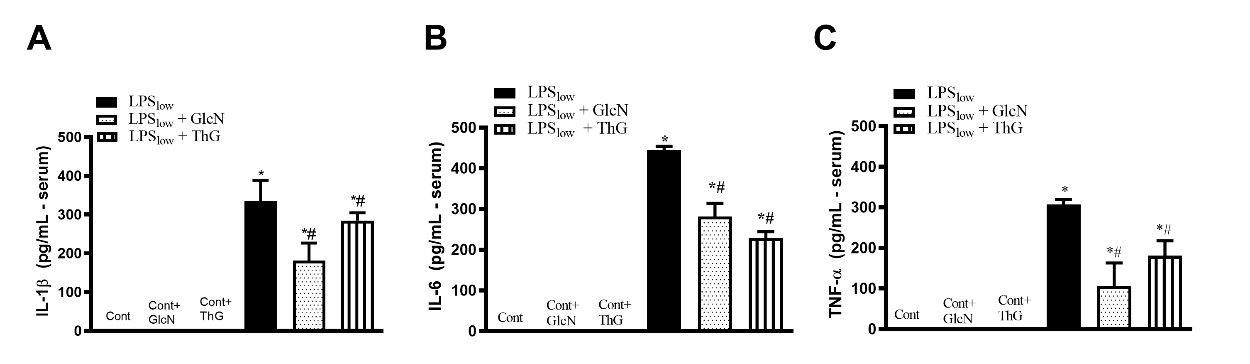


***Supplementary Figure 2: Systemic levels of IL-1β, IL-6, and TNF-α.*** Evaluation of serum concentration of (A) IL-1β, (B) IL-6 and (C) TNF-α in mice submitted to LPS_low_-induced SIRS and treated with vehicle (LPS_low_), GlcN (LPS_low_ + GlcN) or ThG (LPS_low_ + ThG). The results are expressed as mean ± SEM and are representative of 3-4 experiments. *, p<0.05 *vs*. control; #, p<0.05 *vs*. LPS_low_. One-way ANOVA followed by Dunnett's post-test.

Furthermore, treatment of mice with the lower dose of LPS (10 mg/Kg i.p.) also induced a progressive decrease in mean arterial blood pressure (MAP). Although GlcN treatment did not prevent hypotension in mice with LPS_low_, MAP was significantly higher after 5 h of LPS-induced SIRS (Supplementary Figure 3).

**
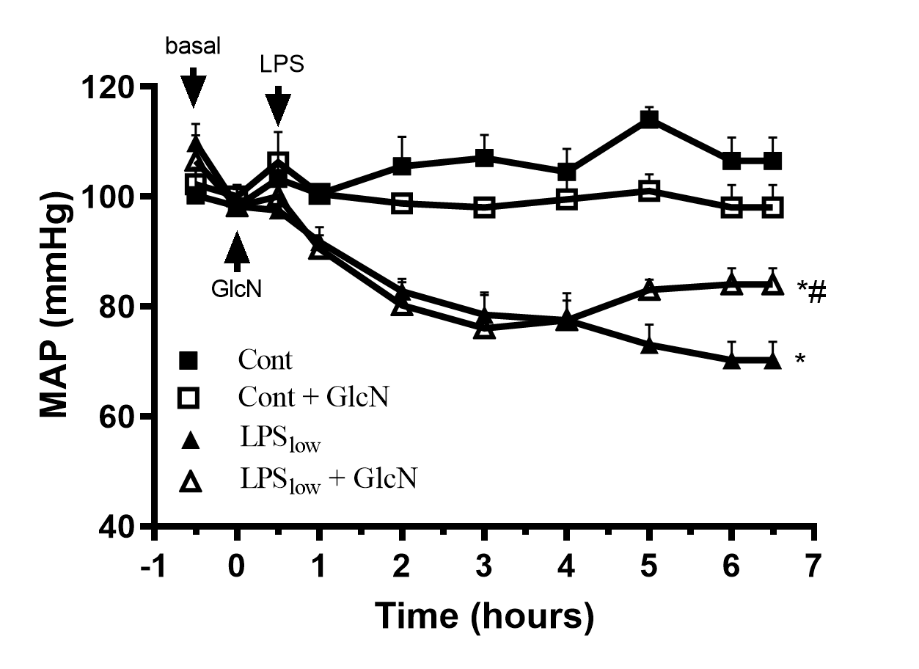
**

***Supplementary Figure 3: Mean arterial pressure (MAP) in LPS mice.*** Mean arterial pressure (MAP) was recorded, via carotid artery cannulation, for 6 h after LPS administration (10 mg/kg, i.p.). GlcN treatment was performed 30 min before LPS injection. The results are expressed as mean ± SEM and are representative of 4 experiments. *, p<0.05 *vs*. control; #, p<0.05 *vs*. LPS_low_. One-way ANOVA followed by Bonferroni’s post-test.

***Vascular reactivity***

The lower LPS dose (10 mg/kg i.p.) reduced mesenteric artery reactivity to phenylephrine (Supplementary Figure 4). In addition, mesenteric arteries from LPS_low_ mice treated with ThG showed increased responses to phenylephrine, indicating improvement of contractile vascular responses. GlcN and ThG treatments did not restore phenylephrine maximal contractile responses (Emax).


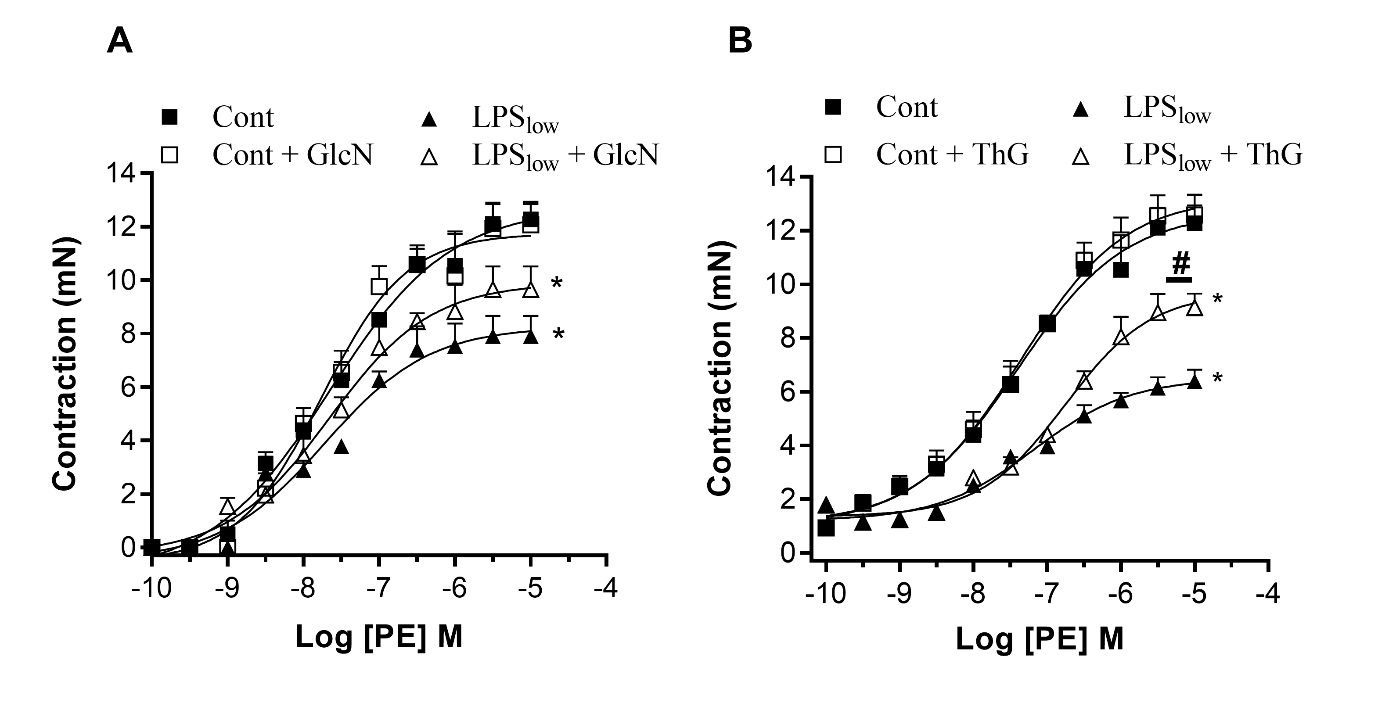


***Supplementary Figure 4:*** Contractile responses to phenylephrine (PE) in mesenteric arteries from control and LPS-induced SIRS (LPS_low_) mice treated with **(A)** vehicle and glucosamine (GlcN) or (**B)** vehicle and thiamet-G (ThG). Data are expressed as mean ± SEM of contraction and are representative of 4-5 experiments. Cont, vehicle-treated control mice; LPS_low_, LPS (10 mg/Kg i.p.)-treated mice; Cont + GlcN and LPS_low_ + GlcN, mice treated with glucosamine (GlcN); Cont + ThG and LPS_low_ + ThG, mice treated with thiamet-G (ThG). *, p <0.05 *vs*. control; #, p <0.05 *vs*. LPS. ANOVA followed by Bonferroni’s post-test.

**Glucosamine reduced CLP- induced circulating IL-6 levels**

To evaluate with GlcN treatment can induce anti-inflammatory effects in septic mice, serum IL-6 level was measured. Antibiotic treatment did not prevent the CLP-induced systemic IL-6 increase. However, when the antibiotic treatment was associated to GlcN, the systemic IL-6 concentration was reduced (Supplementary figure 5).


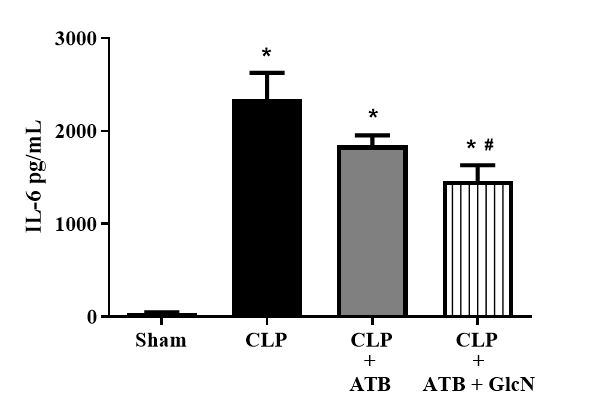


**Supplementary Figure 5:** Systemic IL-6 concentration in control mice (Sham) and mice submitted to cecal ligation and puncture (CLP)-induced sepsis or CLP + ATB (treatment with sodium ertapenem 30 mg/kg s.c., CLP + ATB), or CLP + ATB + GlcN (300 mg/Kg, i.v.). The results are expressed as mean ± SEM and are representative of 4-5 experiments. *, p<0.05 *vs.* sham; #, p<0.05 *vs*. CLP + ATB. One-way ANOVA followed by Dunnett's post-test.
