# Supplementary material for: Acute Increase in O-GlcNAc Improves Survival in Mice With LPS-Induced Systemic Inflammatory Response Syndrome
Source: Front Physiol. 2020 Jan 21;10:1614. doi: 10.3389/fphys.2019.01614 (PMC6985589; doi:10.3389/fphys.2019.01614)
Supplement: Supplementary file 2 [file Presentation_1.PPTX]

## Slide 1
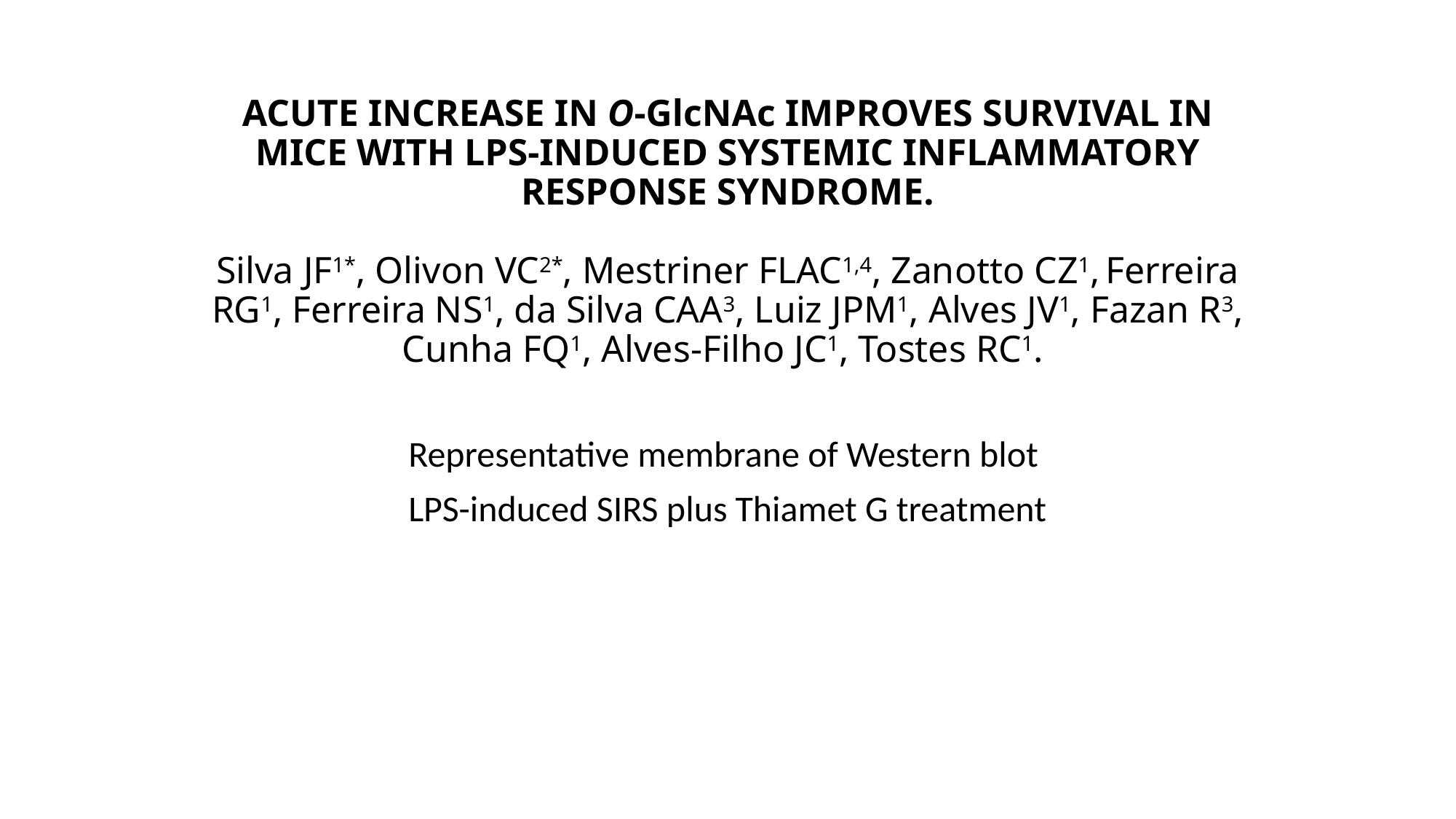

# Acute increase in O-GlcNAc improves SURVIVAL in mice with LPS-induced systemic inflammatory response syndrome.   Silva JF1*, Olivon VC2*, Mestriner FLAC1,4, Zanotto CZ1, Ferreira RG1, Ferreira NS1, da Silva CAA3, Luiz JPM1, Alves JV1, Fazan R3, Cunha FQ1, Alves-Filho JC1, Tostes RC1.
Representative membrane of Western blot
LPS-induced SIRS plus Thiamet G treatment

## Slide 2
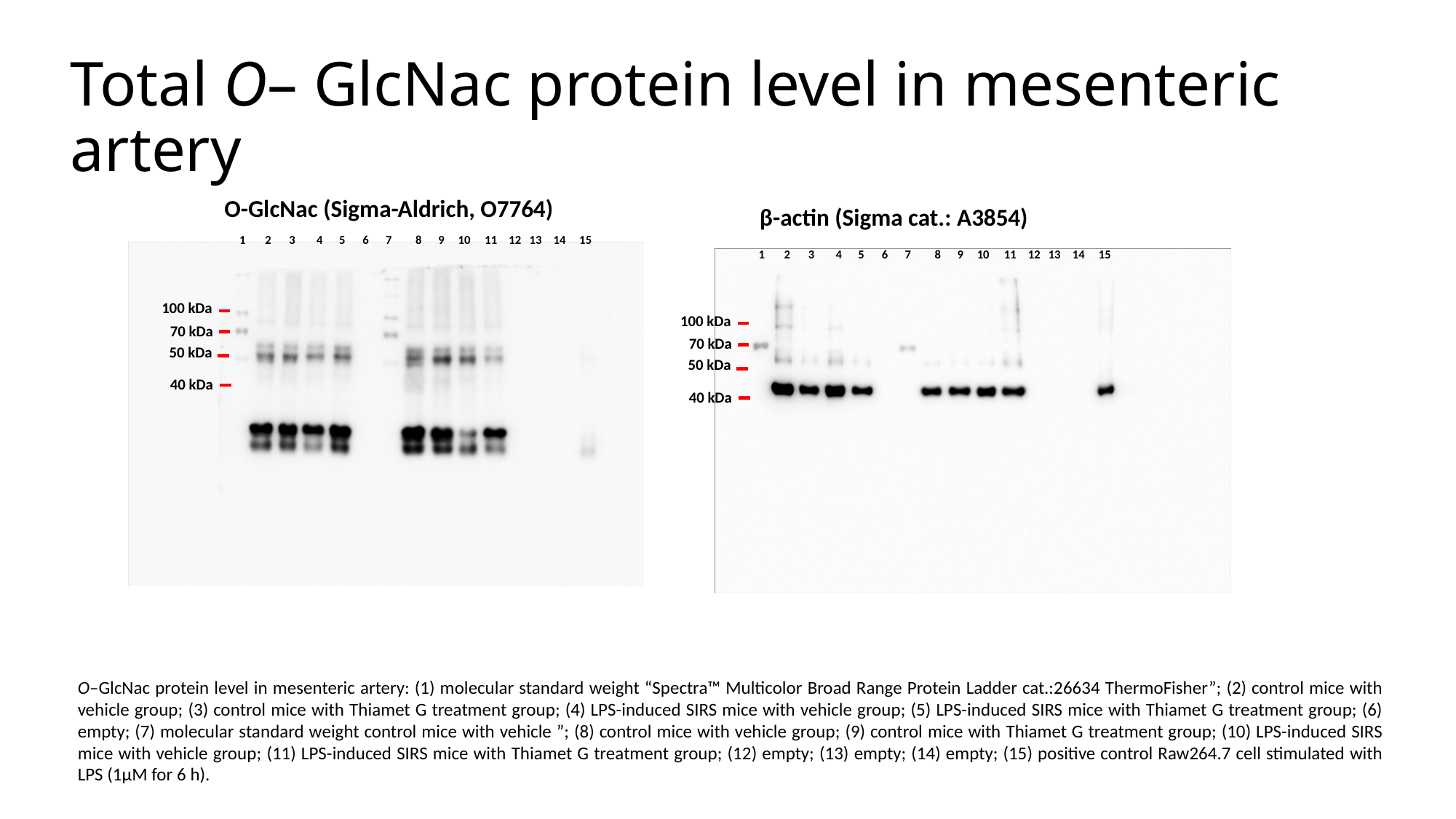

# Total O– GlcNac protein level in mesenteric artery
O-GlcNac (Sigma-Aldrich, O7764)
1
2
3
4
5
6
7
8
9
10
11
12
13
14
15
100 kDa
70 kDa
50 kDa
40 kDa
β-actin (Sigma cat.: A3854)
1
2
3
4
5
6
7
8
9
10
11
12
13
14
15
100 kDa
70 kDa
50 kDa
40 kDa
O–GlcNac protein level in mesenteric artery: (1) molecular standard weight “Spectra™ Multicolor Broad Range Protein Ladder cat.:26634 ThermoFisher”; (2) control mice with vehicle group; (3) control mice with Thiamet G treatment group; (4) LPS-induced SIRS mice with vehicle group; (5) LPS-induced SIRS mice with Thiamet G treatment group; (6) empty; (7) molecular standard weight control mice with vehicle ”; (8) control mice with vehicle group; (9) control mice with Thiamet G treatment group; (10) LPS-induced SIRS mice with vehicle group; (11) LPS-induced SIRS mice with Thiamet G treatment group; (12) empty; (13) empty; (14) empty; (15) positive control Raw264.7 cell stimulated with LPS (1µM for 6 h).

## Slide 3
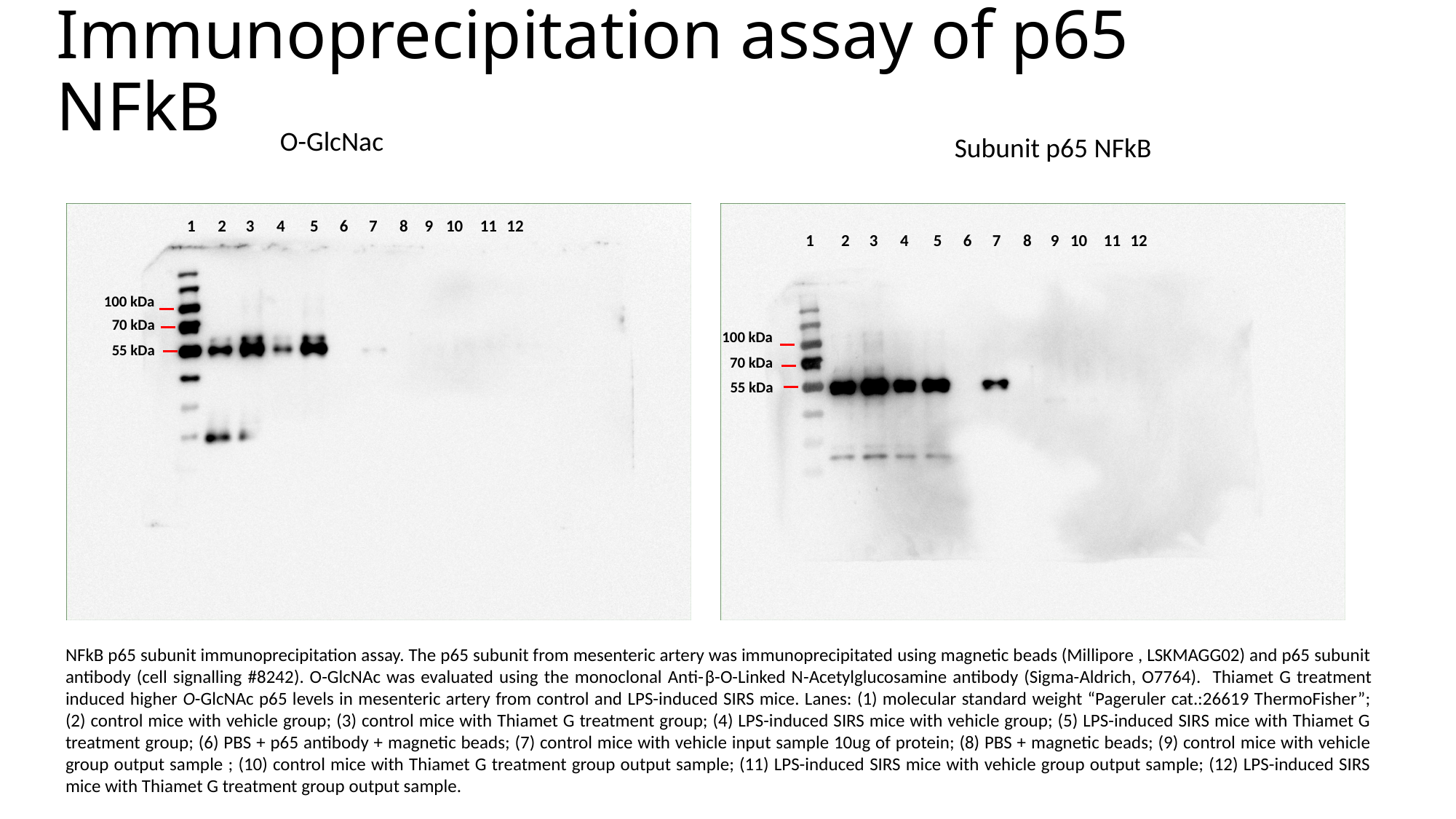

# Immunoprecipitation assay of p65 NFkB
O-GlcNac
1
2
3
4
5
6
7
8
9
10
11
12
100 kDa
70 kDa
55 kDa
Subunit p65 NFkB
1
2
3
4
5
6
7
8
9
10
11
12
100 kDa
70 kDa
55 kDa
NFkB p65 subunit immunoprecipitation assay. The p65 subunit from mesenteric artery was immunoprecipitated using magnetic beads (Millipore , LSKMAGG02) and p65 subunit antibody (cell signalling #8242). O-GlcNAc was evaluated using the monoclonal Anti-β-O-Linked N-Acetylglucosamine antibody (Sigma-Aldrich, O7764). Thiamet G treatment induced higher O-GlcNAc p65 levels in mesenteric artery from control and LPS-induced SIRS mice. Lanes: (1) molecular standard weight “Pageruler cat.:26619 ThermoFisher”; (2) control mice with vehicle group; (3) control mice with Thiamet G treatment group; (4) LPS-induced SIRS mice with vehicle group; (5) LPS-induced SIRS mice with Thiamet G treatment group; (6) PBS + p65 antibody + magnetic beads; (7) control mice with vehicle input sample 10ug of protein; (8) PBS + magnetic beads; (9) control mice with vehicle group output sample ; (10) control mice with Thiamet G treatment group output sample; (11) LPS-induced SIRS mice with vehicle group output sample; (12) LPS-induced SIRS mice with Thiamet G treatment group output sample.

## Slide 4
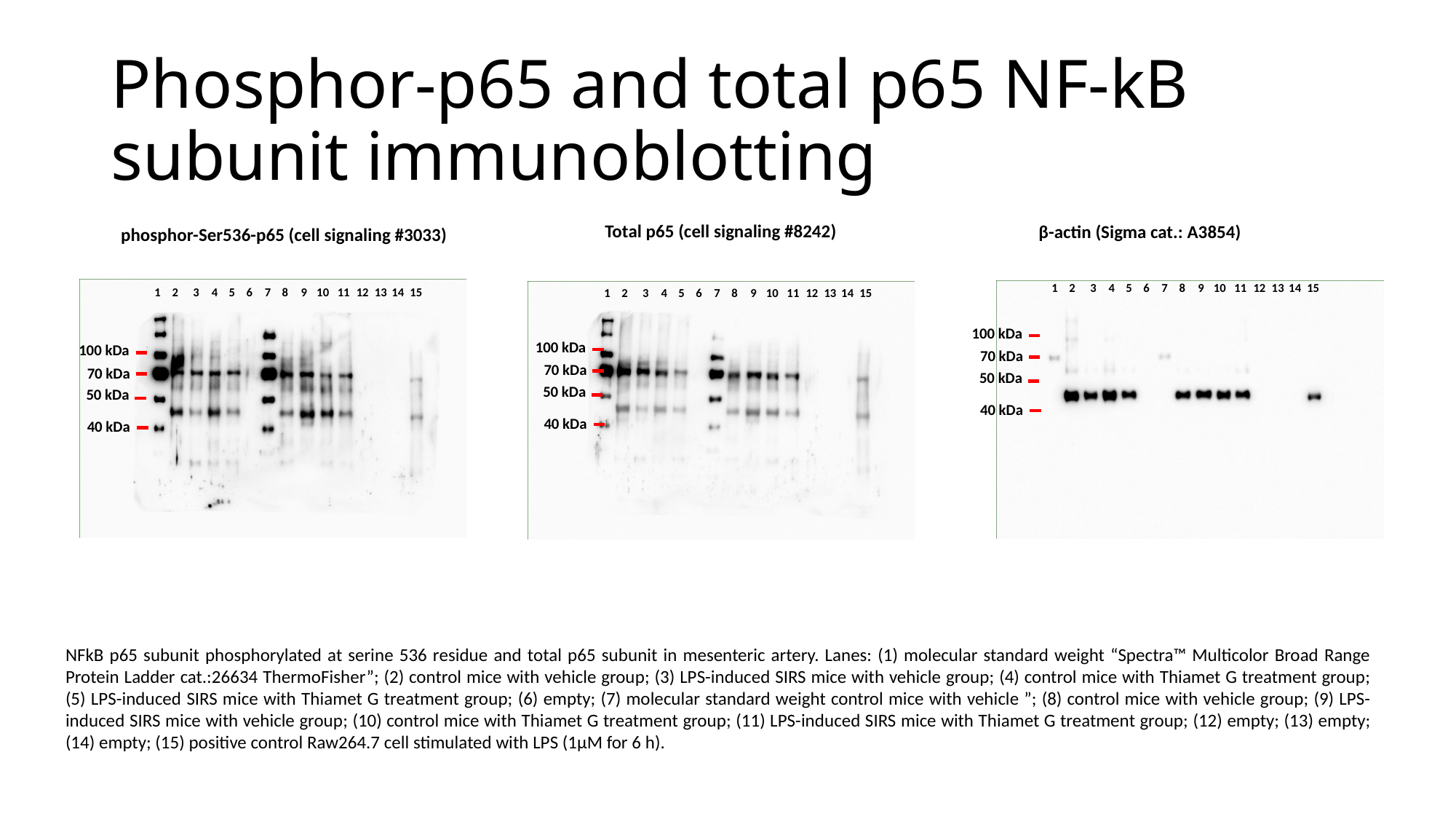

# Phosphor-p65 and total p65 NF-kB subunit immunoblotting
Total p65 (cell signaling #8242)
1
2
3
4
5
6
7
8
9
10
11
12
13
14
15
100 kDa
70 kDa
50 kDa
40 kDa
β-actin (Sigma cat.: A3854)
1
2
3
4
5
6
7
8
9
10
11
12
13
14
15
100 kDa
70 kDa
50 kDa
40 kDa
phosphor-Ser536-p65 (cell signaling #3033)
1
2
3
4
5
6
7
8
9
10
11
12
13
14
15
100 kDa
70 kDa
50 kDa
40 kDa
NFkB p65 subunit phosphorylated at serine 536 residue and total p65 subunit in mesenteric artery. Lanes: (1) molecular standard weight “Spectra™ Multicolor Broad Range Protein Ladder cat.:26634 ThermoFisher”; (2) control mice with vehicle group; (3) LPS-induced SIRS mice with vehicle group; (4) control mice with Thiamet G treatment group; (5) LPS-induced SIRS mice with Thiamet G treatment group; (6) empty; (7) molecular standard weight control mice with vehicle ”; (8) control mice with vehicle group; (9) LPS-induced SIRS mice with vehicle group; (10) control mice with Thiamet G treatment group; (11) LPS-induced SIRS mice with Thiamet G treatment group; (12) empty; (13) empty; (14) empty; (15) positive control Raw264.7 cell stimulated with LPS (1µM for 6 h).
